# Offline Adaptive Radiotherapy for Large Non-small Cell Lung Cancer With Volumetric Modulated Arc Therapy Replanning After Creation of Dose Distribution Using Cone-Beam Computed Tomographic Images of Image-Guided Radiotherapy

**DOI:** 10.7759/cureus.73934

**Published:** 2024-11-18

**Authors:** Takayuki Ohguri, Eiji Shiba, Ryohei Kuroki, Hideaki Matsukawa, Subaru Tani, Yoshio Iwai

**Affiliations:** 1 Therapeutic Radiology, University Hospital of Occupational and Environmental Health, Kitakyushu, JPN; 2 Radiology, University Hospital of Occupational and Environmental Health, Kitakyushu, JPN; 3 Research Physics, Elekta KK, Tokyo, JPN

**Keywords:** adaptive radiotherapy, automated contouring, dose distribution, lung cancer, volumetric modulated arc therapy

## Abstract

There is a paucity of studies reporting the use of automated contouring function on cone-beam computed tomography (CBCT) images acquired during image-guided radiotherapy (IGRT) with offline adaptive radiotherapy for locally advanced non-small cell lung cancer (NSCLC). This case report discusses the use of an automated contouring function on CBCT images acquired during IGRT to quantify the dose distribution variations associated with tumor shrinkage in a patient with large NSCLC. A 72-year-old woman with locally advanced squamous cell carcinoma of the lung (T4N1M0; tumor diameter: 14 cm) underwent chemoradiotherapy. Two offline adaptive radiotherapy sessions were performed, resulting in no evidence of recurrence or distant metastasis, as well as no side effects of Grade 2 or higher toxicity.

## Introduction

Chemoradiotherapy (CRT) remains the primary treatment for locally advanced non-small cell lung cancer (NSCLC). Radiation pneumonitis is a common toxicity associated with thoracic radiotherapy (RT). Several factors, including the total dose, fractionation schedule, and irradiated lung volume, affect the risk of radiation pneumonitis [1]. Furthermore, contemporary studies have suggested that cardiac dose is a significant prognostic factor for overall survival in individuals with locally advanced NSCLC [2]. Intensity-modulated radiation therapy (IMRT)-based CRT is widely used in clinical practice to achieve a high target-dose concentration, while minimizing the dose to at-risk organs, such as the lungs, heart, and esophagus. Furthermore, volumetric modulated arc therapy (VMAT) can significantly reduce treatment time compared to conventional IMRT. VMAT uses a rotating gantry with a variable dose rate and speed, while IMRT uses fixed gantry beams. This approach eases patient burden and improves treatment efficiency. However, VMAT may not effectively deliver the desired target dose to patients with locally advanced NSCLC, especially those with large tumors, while simultaneously minimizing the dose to critical organs such as the lungs, heart, and esophagus.

Adaptive radiotherapy (ART) maximizes the tumor dose and minimizes the dose to healthy tissues by adapting to changes in the tumor and patient’s body during treatment and modifying the treatment plan in real time or at regular intervals. In conventional RT, treatment follows a pretreatment plan, whereas ART adapts to changes in the tumor and the patient’s body using imaging information (from computed tomography (CT) and magnetic resonance imaging (MRI)) to modify the treatment plan, resulting in a more accurate and safer treatment. Moreover, ART has been shown to be more effective in targeting the disease throughout the treatment course in patients with NSCLC [3,4]. Consequently, identifying patients who require ART and determining the optimal timing of treatment initiation are crucial. A recent study showed that cone-beam computed tomography (CBCT) images obtained during image-guided radiotherapy (IGRT) can be used to quantitatively evaluate the changes in dose distribution resulting from tumor shrinkage throughout the treatment course [5]. There is a paucity of studies reporting the use of this technique with offline ART for locally advanced NSCLC. Therefore, this case report describes the use of automatic contouring on CBCT images acquired during IGRT to quantify dose distribution variations associated with tumor shrinkage in a patient with large NSCLC. Two offline ART sessions were performed, resulting in no evidence of recurrence or side effects of Grade 2 severity or higher.

## Case presentation

A 72-year-old woman with locally advanced squamous cell carcinoma of the lung (T4N1M0; tumor diameter: 14 cm) was treated with CRT. The patient had a history of hypertrophic cardiomyopathy, chronic heart failure, and chronic kidney disease. In addition, the patient had a history of medically managed type B aortic dissection. Because of the large primary tumor and preexisting heart disease, it was necessary to reduce the doses to the healthy lung and heart, while delivering the curative dose to the target organ. The treatment device was Versa HD (Elekta AB, Stockholm, Sweden) with Monaco ver. 5.5 (Elekta AB, Stockholm, Sweden) treatment planning device. The dose distribution could be improved by re-planning VMAT according to the reduction in tumor size. Therefore, we decided to use CBCT for position matching to calculate dose distribution and determine the timing of offline ART. The target dose fractionation of the VMAT plan was 60 Gy in 30 fractions. The initial plan started with planning target volume (PTV D95) of 58.3 Gy, lung V20 of 27.5%, lung V5 of 37.5%, and heart Dmean of 9.2 Gy. Concurrent chemotherapies were administered at a reduced dose given the patient’s underlying medical conditions. Carboplatin, with an area under the curve of 1.2, and 22.5 mg/m² paclitaxel were administered weekly throughout the course of RT.

**Figure 1:**
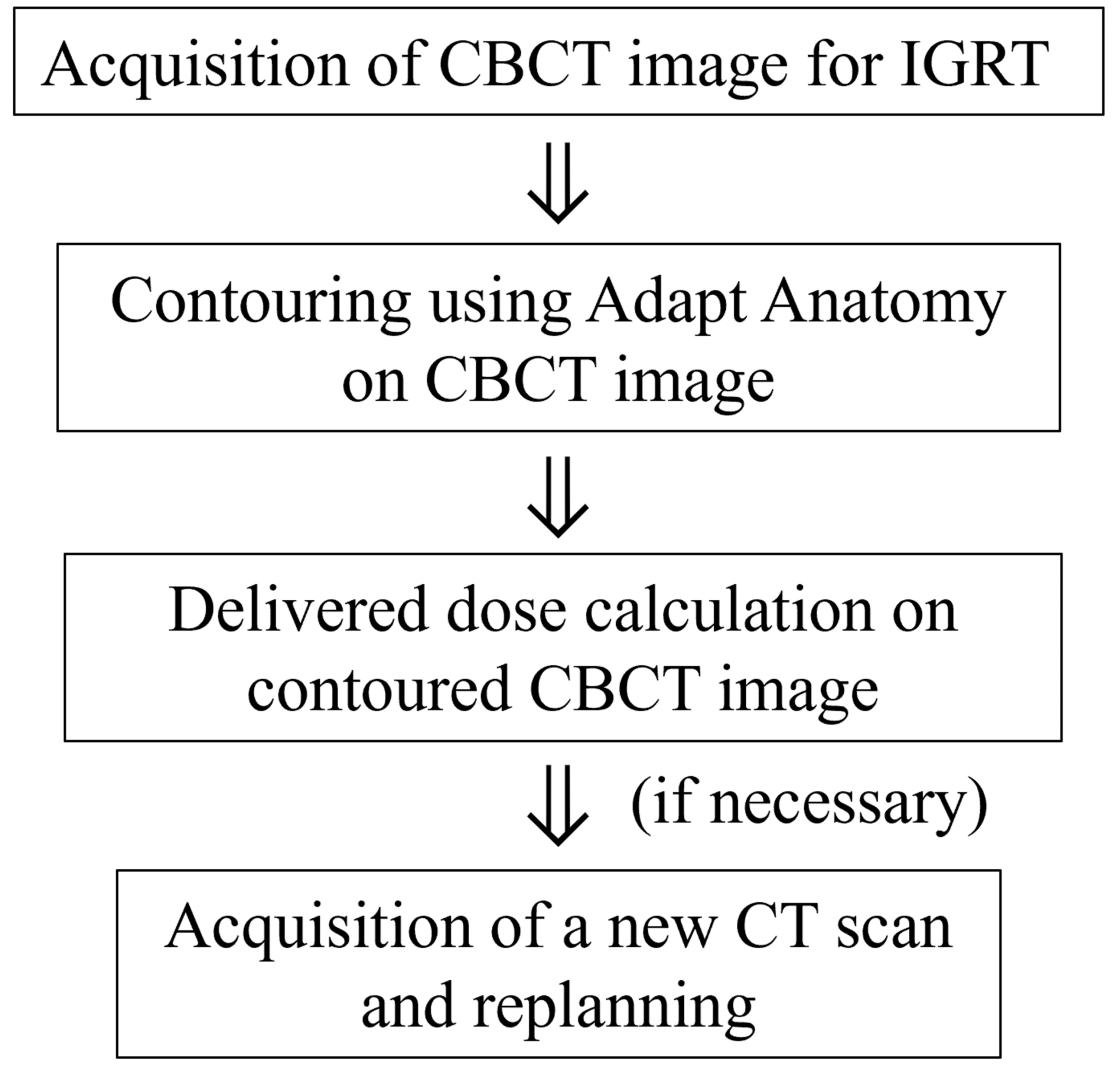
Schema of our workflow for offline ART. ART: adaptive radiotherapy; CBCT: cone-beam computed tomography; DVH: dose-volume histogram; IGRT: image-guided radiotheraphy

Our unique workflow for offline ART, which incorporates adaptation to changes in the tumor and the patient’s body during treatment, is illustrated in Figure 1.

**Figure 2:**
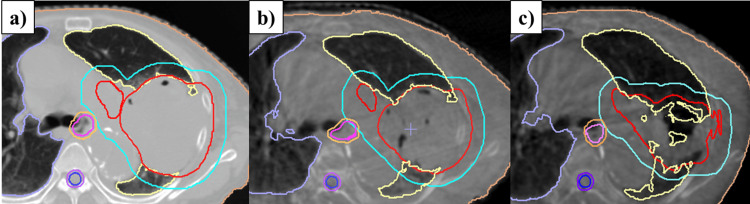
Adapt Anatomy on CBCT images. (a) Initial plan contouring. (b) Automatic contouring using Adapt Anatomy at the time of 20 Gy CBCT and (c) 40 Gy CBCT. The GTV is shown in red, the PTV in light blue, the esophagus in magenta, the spinal cord in blue, the left lung in light yellow, and the right lung in light purple. CBCT: cone-beam computed tomography; GTV: gross tumor volume; PTV: planning target volume

Adapt Anatomy implemented in the Monaco ver. 5.5 treatment planning device was used to automatically generate structures for CBCT based on the structures of the initial planning CT. Dose calculations were performed for each structure using electron density information from the planning CT, and dose distributions were generated. Because of the patient’s small physique (height: 147 cm, weight: 44 kg), the entire lung could be imaged with CBCT for IGRT, and the CBCT-based dose distribution at 20 Gy showed a lung V20 of 31.1%, lung V5 of 40.8%, and PTV D95 of 59.2 Gy, indicating an increase in lung dose parameters due to tumor shrinkage and increased normal lung volume. After re-planning CT imaging and re-planning after 24 Gy, irradiation was continued with a combined dose of lung V20 of 27.8%, lung V5 of 34.6%, and PTV D95 of 61.1 Gy. The tumor volume was further reduced, and the CBCT dose distribution at 42 Gy showed lung V20 of 29.0%, lung V5 of 37.6%, and PTV D95 of 61.0 Gy, again indicating an increase in the lung dose parameters. Therefore, a third planning CT scan was performed. After achieving 48 Gy, the third plan was implemented and the treatment was completed at 60 Gy in 30 fractions. Figure 2a shows a contour image of the initial treatment plan. Figure 2b and Figure 2c show the contour images created using Adapt Anatomy on the CBCT images at 20 Gy and 40 Gy, respectively.

**Figure 3:**
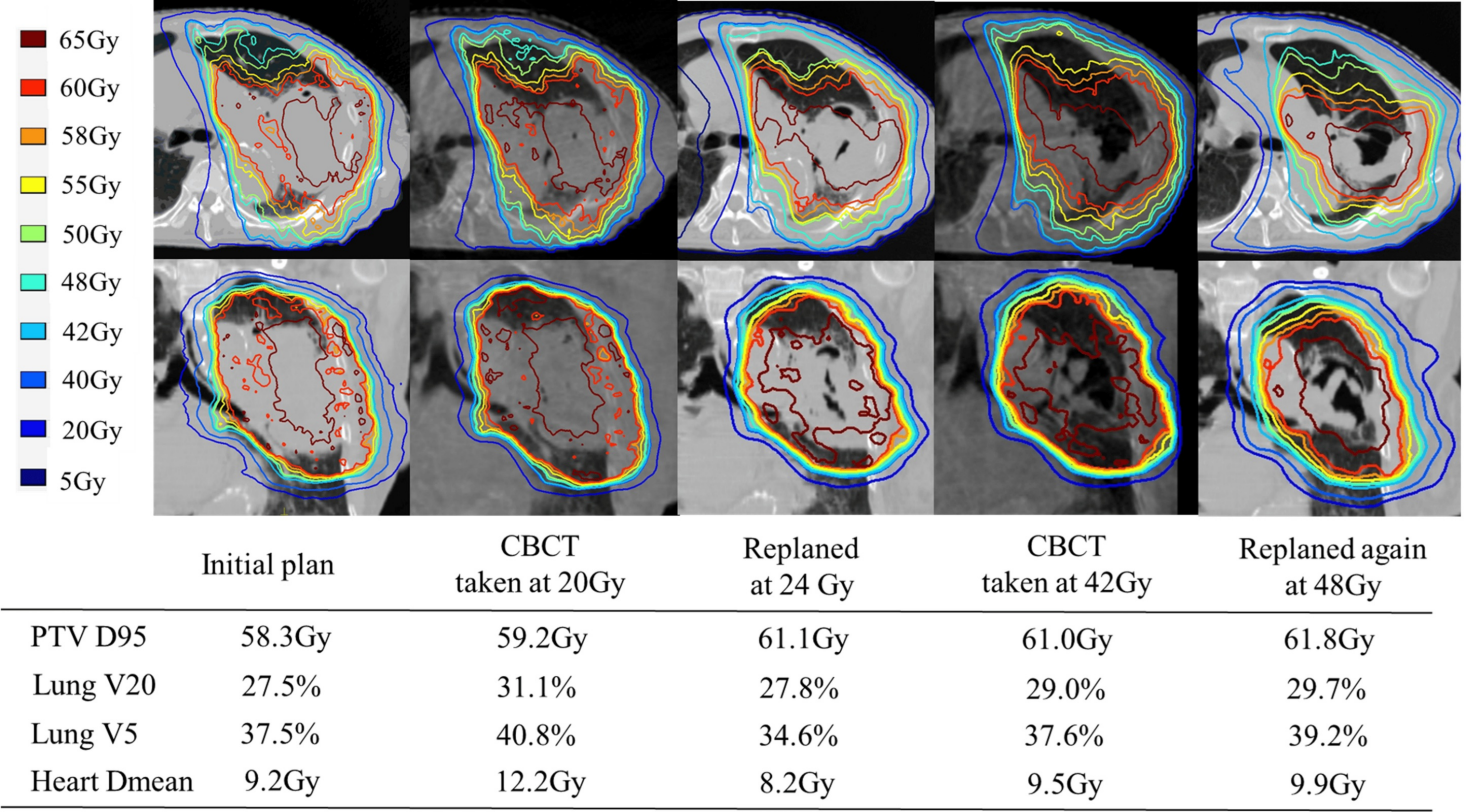
Dose distribution diagrams, including PTV dose and doses to at-risk organs, at each time point. CBCT: cone-beam computed tomography; PTV: planning target volume

Figure 3 shows the dose distribution maps of the axial and coronal planes at each time point, as well as the PTV dose and dose parameters for the organs at risk.

**Figure 4:**
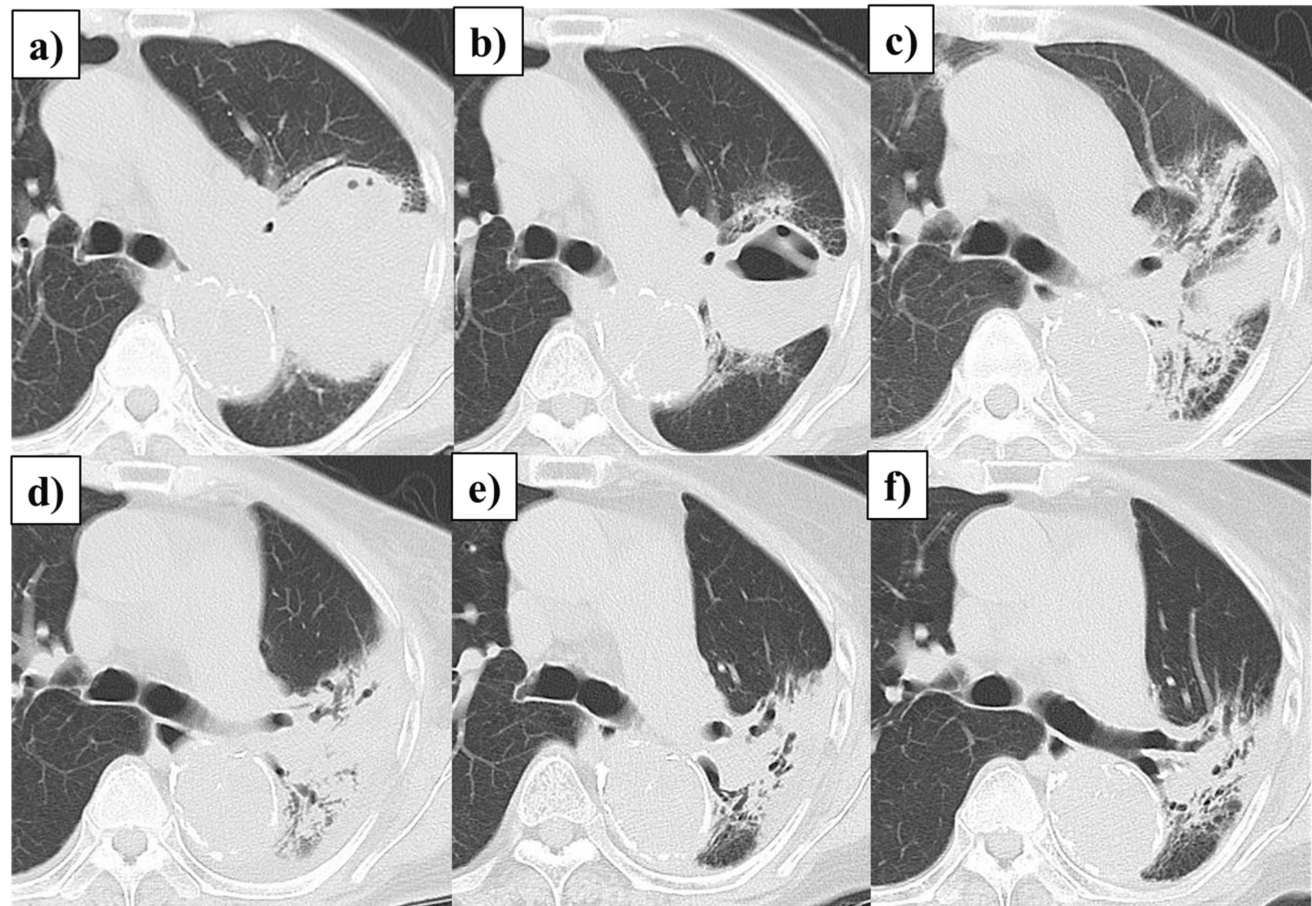
Serial CT images showing treatment response. (a) Baseline CT before the initiation of concurrent chemoradiotherapy. (b) One-month post-radiotherapy: tumor reduction. (c) Three months: Grade 1 radiation pneumonitis. (d) Seven months: improvement in radiation pneumonitis. (e) Thirteen months: continued tumor reduction. (f) Thirty-three months: persistent tumor reduction. CT: computed tomography

No acute side effects of Grade 2 or higher were observed. Figure 4 shows the patient’s progress after the completion of RT.

Following the completion of RT, durvalumab was administered every two weeks at a dose of 10 mg/kg for four cycles. Grade 1 radiation pneumonitis was observed; 33 months after completion of RT, there was no evidence of recurrence or distant metastasis.

## Discussion

This case report demonstrates a successful application of offline ART by evaluating dose distribution changes using the automatic contouring function of CBCT images acquired during IGRT. Two offline re-plannings were implemented, leading to improved dose parameters and a favorable outcome. ART can be classified into offline and online modalities. Offline ART typically addresses systematic variations, while online ART accounts for both systematic and random variations. However, online ART requires a significant amount of effort online while the patient lies on the treatment table. Our offline ART workflow is a simple method for quantifying changes in dose distribution using CBCT for IGRT and examining the timing of ART implementation. To our knowledge, there is a paucity of literature reporting the use of this technique with offline ART for locally advanced NSCLC.

ART enables physicians to reduce the target volume in response to tumor shrinkage during treatment, potentially mitigating acute and long-term treatment-related toxicity. The clinical efficacy of this approach hinges on the temporal and quantitative aspects of tumor volume changes throughout treatment. RT for locally advanced NSCLC has been demonstrated to significantly reduce primary tumor volume during treatment. Wald et al. reported primary tumor volume changes as measured by kV-CBCT during definitive CRT for stage III NSCLC; within two weeks of treatment, 50% of patients exhibited a relative tumor reduction exceeding 30%, indicating that a substantial number of patients may benefit from adaptive replanning [6]. A Phase 2 clinical trial evaluating concurrent chemo-proton therapy for patients with unresectable stage III NSCLC, incorporating offline adaptive planning, was reported. Adaptive planning was implemented, with CT scans obtained at 10, 20, and 30 days following treatment initiation to inform replanning decisions [7]. With a median of 2.5 replanning sessions per patient, the five-year local control rate reached 61%. Toxicity was generally well-managed, with no Grade 3 or higher radiation pneumonitis observed. Only 2% of patients experienced Grade 3 heart failure. Recent studies have shown a link between increased cardiac radiation exposure and a higher risk of heart problems. Specifically, in cases where the average heart dose was 20 Gy or more, the four-year incidence of symptomatic coronary events was significantly higher in patients with coronary artery calcification (60%) compared to those without calcification (13%) [8]. Reducing the heart dose is essential for the future of thoracic RT. ART and other advanced techniques are expected to play a key role in achieving this goal.

In our case, CBCT-based daily position matching and the automatic contouring function allowed for straightforward assessment of variations in target size, position, and normal lung dose, enabling timely decisions regarding offline replanning. This case involved a large tumor measuring 14 cm. Despite using VMAT, it was challenging to meet normal lung dose constraints. The prescribed dose to the PTV was 60 Gy at D95. Initially, D95 was reduced to 58.3 Gy to limit lung V20 to 27%. Recent studies on concurrent chemoradiotherapy for stage III NSCLC have shown a higher incidence of radiation pneumonitis in Asian patients [9]. To prevent symptomatic radiation pneumonitis in Asian patients, the lung V20 should be limited to 25-30% or less, and the lung V5 to 45% or less. [10,11]. Two times re-plannings ensured that the PTV D95 remained at 60 Gy while controlling the increase in lung V20 to less than 30% and ling V5 to less than 45%. ​​​​​​​This was crucial as the tumor shrank and normal lung tissue expanded. Additionally, the patient had a serious heart condition, raising concerns about increased cardiovascular risk due to the elevated heart dose. However, the average heart dose was successfully maintained below 10 Gy.​​​​​​​ ART can be implemented through the following two main strategies: (1) sparing organs at risk while maintaining the PTV dose (the isoeffective scenario) and (2) escalating the PTV dose without increasing the organs-at-risk dose (the isotoxic scenario). In our second replanning, the priority was to improve PTV D95 coverage while maintaining the organs-at-risk dose.

## Conclusions

Here, we presented a case of successful treatment of locally advanced NSCLC, in which the timing of offline ART was evaluated using the automatic contouring function of CBCT images acquired under IGRT. Further studies are warranted to evaluate the efficacy of ART using this method in patients with locally advanced NSCLC who exhibit changes in target size, position, or organs at risk during RT.
